# Adoption of Machine Learning in US Hospital Electronic Health Record Systems: Retrospective Observational Study

**DOI:** 10.2196/76126

**Published:** 2025-12-09

**Authors:** Huang Huang, Wei Lyu, Md Mahmud Hasan, Shannon H Houser

**Affiliations:** 1 Department of Health Management, Economics and Policy, School of Public Health, Augusta University, 2500 Walton Way, Science Hall, E-1031, Augusta, GA, 30904, United States, 1 8595519185; 2 Department of Health Services Administration, University of Alabama at Birmingham, Birmingham, AL, United States; 3 Department of Biostatistics, Data Science and Epidemiology, School of Public Health, Augusta University, Augusta, GA, United States

**Keywords:** artificial intelligence, electronic health record, health information technology adoption, machine learning, organizational behavior

## Abstract

**Background:**

While machine learning (ML) technologies have shifted from development to real-world deployment over the past decade, US health care providers and hospital administrators have increasingly embraced ML, particularly through its integration with electronic health record (EHR) systems. This evolving landscape underscores the need for empirical evidence on ML adoption and its determinants; however, the relationship between hospital characteristics and ML integration within EHR systems remains insufficiently explored.

**Objective:**

This study aimed to examine the current state of ML adoption within EHR systems across US general acute care hospitals and to identify hospital characteristics associated with ML implementation.

**Methods:**

We used linked data between the 2022‐2023 American Hospital Association Annual Survey and the 2023‐2024 American Hospital Association Information Technology Supplement Survey. The sample includes 2562 general and acute care hospitals in the United States with a total of 4055 observations over 2 years. Applying inverse probability weighting to address nonresponse bias, we used descriptive statistics to assess ML adoption patterns and multivariate logistic regression models to identify hospital characteristics associated with ML adoption.

**Results:**

Overall, about 75% of the hospitals had adopted ML functions within their EHR systems in 2023‐2024, and the majority tended to adopt both clinical and operational ML functions simultaneously. The most commonly adopted individual functions were predicting inpatient risks and outpatient follow-ups. ML model evaluation practices, while still limited overall, showed notable improvement. Multivariate regression estimates indicate that hospitals were more likely to adopt any ML if they were not-for-profit (4.4 percentage points, 95% CI 0.6-8.2; *P*=.02), large hospitals (15 percentage points, 95% CI 9.4-21; *P*<.001), operated in metropolitan areas (4.3 percentage points, 95% CI 0.8-7.8; *P*=.02), contracted with leading EHR vendors (20.6 percentage points, 95% CI 17.1-24; *P*<.001), and affiliated with a health system (26.8 percentage points, 95% CI 22.4-31.3; *P*<.001). Similar patterns were observed for predicting the adoption of both clinical and operative ML. We also identified specific hospital characteristics associated with the adoption of individual ML functions.

**Conclusions:**

ML adoption in hospitals is influenced by organizational resources and strategic priorities, raising concerns about potential digital inequities. Limited quality control and evaluation practices highlight the need for stronger regulatory oversight and targeted support for underresourced hospitals. As the integration of ML into EHR systems expands, disparities in both adoption and oversight become increasingly critical. To ensure the equitable, safe, and effective implementation of ML technologies in health care, well-designed policies must address these gaps and promote inclusive innovation across all hospital settings.

## Introduction

Machine learning (ML) has significant potential to enhance diagnostic accuracy, support clinical decision-making, and facilitate early disease detection [1-7]. Additionally, contemporary ML technologies that streamline administrative tasks, refine billing and coding workflows, and enhance patient flow may substantially benefit hospital operations, particularly in underserved areas facing workforce shortages [8-11]. As recent ML advancements shift the focus from development to deployment, US health care providers have increasingly embraced these technologies [12-14], especially through integration with electronic health records (EHRs) [15-18]. Integrating ML techniques into EHRs offers multiple advantages such as simplifying data sharing, reducing analytical time, and mitigating the risk of data leakage [19]. These techniques enable the efficient processing and analysis of vast amounts of unstructured EHR data, which is critical for research but remains largely underutilized [20]. Additionally, ML-driven automation can help alleviate provider burnout by reducing manual data entry, minimizing human errors, and shortening documentation time [21,22].

Despite significant benefits, several technical and ethical challenges (eg, patient privacy, model accuracy, and data reliability) associated with ML adoption may result in unanticipated adverse consequences for patients [2,23-26]. Moreover, the implementation of new health technology is often complicated by various social and organizational factors, posing potential risks to care delivery [23,24]. For instance, hospitals serving more vulnerable populations may lack adequate technical support or sufficient staff when implementing ML in EHRs [27]. Thus, both the benefits and challenges warrant further exploration of the factors influencing ML adoption, as hospitals continue to enhance clinical outcomes and operational efficiency by embracing new health technologies.

Although prior work has examined the implementation of ML in hospital settings [28-30], most studies concentrated on overall adoption, typically measured as the presence of any ML functions or the number of ML functions adopted in EHRs. Such an oversimplified classification treats ML-enabled tools as a homogeneous technology and obscures substantial variation across ML functions. For instance, ML for patient scheduling and ML for inpatient risk prediction differ markedly in data requirements, implementation complexity, and regulatory oversight [31-33]. Prior studies may mask meaningful heterogeneity in real-world ML implementation. Moreover, hospital adoption patterns may vary across particular ML domains or functions depending on distinct organizational needs, resource capacities, and technological benefits (eg, a large academic hospital may prioritize ML for clinical decision support, while a smaller facility may favor administrative automation). Prior research focusing solely on overall adoption may fail to capture this nuance. To address these gaps and better reflect the complexity of ML diffusion in real-world hospitals, our study differentiates 3 dimensions of adoption: (1) overall ML adoption, (2) domain-specific ML adoption at different levels, and (3) adoption of individual ML functions. This multidimensional approach yields a more granular understanding of how hospitals implement ML within EHRs and how adoption patterns differ by institutional context.

In this study, our goals are to (1) describe the status quo of ML adoption in EHRs across US general acute hospitals and (2) identify hospitals and characteristics associated with ML adoption in EHRs, guided by the Technology-Organization-Environment (TOE) framework. Leveraging a national hospital sample and regression analysis, our study offers valuable insights for policymakers, health care administrators, and clinicians regarding the current landscape of ML adoption and for identifying strategies to navigate the opportunities and challenges posed by emerging technologies.

## Methods

### Conceptual Framework

In this study, we integrated the TOE framework with our regression analysis to explain the characteristics that are associated with the ML adoption in EHRs [34,35]. TOE suggests that ML adoption decisions are shaped by 3 contexts: technology, organization, and external environment. The technological context reflects technical benefits and costs of the current ML innovation, such as ML relative advantage, implementation complexity, compatibility with existing EHR systems and workflows, and technical maturity. The organizational context captures hospital internal readiness and capability, including hospital size and structure, leadership support, IT staffing, available financial resources, and IT-supportive organizational culture. Environmental context encompasses all external enablers or barriers outside hospitals such as market competition, artificial intelligence (AI)–related regulations and policy, or EHR vendor support. In health care settings, TOE has been widely used to explain health technology adoption in hospitals [36-38]. These characteristics in TOE offer inevitable clues of understanding who adopts, who benefits, and where digital capability gaps persist. Recognizing these factors in the adoption of ML technology is critical for designing future policies and practice interventions that promote equitable and effective ML implementation across the US health care system.

### Data and Sample

We utilized data from the 2022‐2023 American Hospital Association (AHA) Annual Survey and the 2023‐2024 AHA IT Supplement Survey to examine the relationship between hospital characteristics and ML adoption into EHR [39]. Each year, the AHA surveys health care administrators from 6200 US hospitals and 400 health care systems, collecting information on hospital demographics, operations, and financial metrics. In addition to the main survey, the AHA conducts the IT Supplement Survey, which gathers data on health IT adoption, tools, and barriers across several domains, including patient engagement, social determinants of health, health information exchange, EHR systems, and IT vendors. The IT Supplement Survey is typically completed by the chief information officer, and participation is voluntary. Beginning in 2023, the IT Supplement introduced a series of questions on ML and other predictive models, enabling the investigation of various aspects of ML adoption, such as functionality, developers, and model evaluation practices.

Our study cohort comprises US general and acute care hospitals operating across all 50 states and the District of Columbia that successfully responded to the 2023‐2024 AHA IT Supplement Survey. After applying pairwise deletion for missing data and implementing other exclusion criteria, the final analytic sample includes 2562 unique hospitals across multiple years, resulting in a total of 4055 hospital-year observations.

### ML Measures

ML adoption is measured based on 3 sets of outcomes using the survey questions from the AHA IT Supplement. The first outcome is a binary indicator for any ML adoption, which equals 1 if a hospital uses any ML or other predictive models. Second, to capture the specific uses of this ML, we used a follow-up question to create a 4-category measure. This question asked respondents to select from a list of specific ML applications, which we grouped into 2 domains. Clinical functions included (1) predicting health trajectories or risks for inpatients, such as early detection of conditions like sepsis or in-hospital fall risk; (2) identifying high-risk outpatients to inform follow-up care (eg, readmission risk); (3) monitoring health through integration with wearable devices; and (4) recommending treatments by identifying similar patients and their outcomes. Operational functions included (5) simplifying or automating billing procedures and (6) facilitating scheduling, such as predicting no-shows or optimizing block utilization. Based on both domains, we classified hospitals’ ML use into 1 of 4 mutually exclusive categories: (1) no ML adoption, (2) adoption of clinical functions only, (3) adoption of operational functions only, and (4) adoption of both. This categorical outcome allows for a more nuanced analysis of a hospital’s implementation strategy and provides an interpretable proxy for its organizational readiness. Open-ended responses for other functions were excluded due to their low frequency. Finally, we also analyzed the adoption of individual ML functions listed above.

### Hospital and Environmental Factors

Inspired by the TOE framework and prior studies of health IT adoption [40], we selected the following hospital characteristics as explanatory variables. For organizational context, we included hospital ownership (defined as nonfederal governmental, not-for-profit, and for-profit), bed size (classified as small [0‐99 beds], medium [100‐399 beds], and large [400 or more beds]), critical access hospital (CAH) status, and teaching hospital status. Regarding environmental context, we included health system affiliation (defined as whether hospitals are affiliated with health systems), whether to contract with leading EHR vendors which is identified by EHR market share [41], and metropolitan location (ie, if a hospital is located in a county identified by Rural-Urban Continuum Codes Category 1‐3, it is defined as a metropolitan hospital, otherwise a nonmetropolitan hospital). Due to the lack of relevant technical context information in the AHA survey, this study did not include the technological context.

### Statistical Analysis

First, we reported descriptive statistics for ML adoption, specific ML functions adopted in EHR, who developed ML in EHR, and how hospitals evaluated ML models (ie, accuracy, bias, and postimplementation). Second, we summarized ML adoption status by different hospital characteristics. Chi-squared tests were performed to determine statistically significant differences between hospitals with and without ML adoption.

We further assessed the associations between hospital characteristics and ML adoption using multivariate regressions. We used logistic regressions for ML adoption and multinomial logistic regression for a 4-category measure of ML adoption type. Besides explanatory variables listed above, all regressions controlled for year and geographic region based on Census Divisions (ie, New England, Middle Atlantic, East North Central, West North Central, South Atlantic, East South Central, West South Central, Mountain, and Pacific). For all outcomes, we report marginal effects with 95% CIs, which can be directly interpreted as changes in percentage points in the likelihood for a specific category of ML adoption [42,43]. SEs are clustered at the hospital level to account for a within-hospital correlation over time. We consider estimates to be statistically significant at *P*<.05. All statistical analyses are performed using StataNow/SE 18.5 version [44].

Given high nonresponse rates in the AHA IT Supplement, we compared the characteristics of respondents versus nonrespondents and found that nonrespondent hospitals were more likely to be smaller, less system affiliated, and located in rural areas—factors which are potentially associated with lower adoption rates (Table S1 in Multimedia Appendix 1). To address potential nonresponse bias and produce nationally representative estimates, we constructed inverse probability weights (IPWs) from the regression that estimated each hospital’s probability of responding to the AHA IT Supplement (propensity score) using the same set of characteristics described above as predictors [45,46]. IPW mitigates selection bias by reweighting observations based on their propensity to respond to the AHA IT Supplement, thereby creating a pseudo-population where the distribution of observed covariates is balanced between respondents and nonrespondents. Specifically, for each year, we fitted a separate logistic regression model and predicted the probability of responding to the AHA IT Supplement. We then constructed the analysis weights as the inverse of this predicted response probability and applied these weights in all our statistical analyses. After adjusting by IPWs, the previously observed discrepancies between respondents and nonrespondents became smaller (Table S1 in Multimedia Appendix 1), which suggests mitigating the nonresponse bias. All descriptive analyses and regressions are weighted using the IPWs. It is important to note that this IPW approach assumes that nonresponse depends only on observables; accordingly, it corrects for bias related to the measured hospital characteristics. However, any unobserved factors correlated with both survey nonresponse and ML adoption could still bias our estimates, which could not be fully addressed in our study.

### Ethical Considerations

Our study is a secondary analysis of organization-level data whose original survey design and administration are described in AHA technical documentation [39], and does not involve human participants. Under the US Common Rule (45 CFR §46), research using deidentified, organization-level data does not constitute human subjects research and therefore does not require Institutional Review Board oversight [47]. We accessed the AHA data via Wharton Research Data Services (WRDS), under data sharing agreements between the AHA and WRDS and between WRDS and the University of Alabama at Birmingham. The results are reported in accordance with the Strengthening the Reporting of Observational Studies in Epidemiology (STROBE) guidelines.

## Results

### Descriptive Analysis

#### Overall ML Adoption

The flowchart of our sample selection is presented in Figure S1 in Multimedia Appendix 1. Among our 2023‐2024 analytical sample, 73%‐76% of the hospitals reported any ML functionality within their EHR system (Figure S2 in Multimedia Appendix 1). The majority of US hospitals tend to adopt both clinical and operative ML functions, while the share increased by 10.7 percentage points from 2023 to 2024.

As summarized in Table 1, 83.8% of the metropolitan hospitals adopted ML in EHRs, while only 61.3% of the nonmetropolitan hospitals adopted MLs (all subsequent percentages follow the same interpretation). Adoption rates were higher among large hospitals (94.4% vs 65.0% for small), those contracted with leading EHR vendors (92.1% vs 56.7% for nonleading EHR), not-for-profit hospitals (82.7% vs 69.8% for for-profit), non-CAHs (82.2% vs 57.4% for CAHs), those affiliated with health systems (87.9% vs 40.1% for non-health-system), and teaching hospitals (92.1% vs 56.7% for non-teaching). The geographic distribution of any ML adoption is exhibited in Figure S3 in Multimedia Appendix 1.

**Table 1.** ML^a^ adoption in electronic health record systems by hospital characteristics^b^.

	Without ML (%)	With ML (%)	*P* value (*χ*^2^ test)
Organizational context			
Hospital type			<.001
Nonfederal, governmental	48.6	51.4	
Not-for-profit	17.4	82.7	
For profit	30.2	69.8	
Hospital size			<.001
Small, 0‐99 beds	35.0	65.0	
Medium, 100‐399 beds	17.9	82.1	
Large, 400 or more beds	5.6	94.4	
Critical access hospital			<.001
No	17.8	82.2	
Yes	42.6	57.4	
Teaching status			<.001
No	25.8	74.2	
Yes	6.7	93.3	
Environmental context			
Health system			<.001
No	59.9	40.1	
Yes	12.1	87.9	
Leading EHR^c^^,^^d^			<.001
No	43.3	56.7	
Yes	7.9	92.1	
Metropolitan^e^			<.001
No	38.7	61.3	
Yes	16.2	83.8	
Region			<.001
New England	27.3	72.7	
Middle Atlantic	20.3	79.7	
East North Central	23.8	76.2	
West North Central	26.3	73.7	
South Atlantic	11.6	88.4	
East South Central	21.8	78.6	
West South Central	40.7	59.3	
Mountain	26.6	73.4	
Number of hospital-year observations	825	3230	

a ML: machine learning.

b The data are from the 2022‐2023 American Hospital Association (AHA) Annual Survey and 2023‐2024 AHA IT Supplement. Percentages are weighted using propensity-score inverse-probability weights to adjust for nonresponse to the IT Supplement.

c Leading EHR vendors are identified by market share.

d EHR: electronic health record.

e Metropolitan status is categorized based on the 2023 Rural-Urban Continuum Codes: 1‐3 as metropolitan counties and 4‐9 as nonmetropolitan counties.

#### Specific ML Function

The most widely adopted functions (Figure S4 in Multimedia Appendix 1) are predicting health trajectories or inpatient risks and identifying high-risk outpatients for follow-up care. From 2023 to 2024, the adoption of clinical ML functions remained stable, whereas operational functions rose markedly, specifically by 19.9 percentage points for simplifying or automating billing procedures and by 14.3 percentage points for facilitating scheduling.

#### ML Development

Hospitals leveraged multiple resources to develop ML or other predictive models (Figure S5 in Multimedia Appendix 1). About 70%‐80% of hospitals reported that their ML was developed by their EHR vendors. The third-party developers and in-house IT teams continued to play meaningful roles in ML development, which likely reflects hospitals’ needs in customizing features to meet varying clinical and administrative needs. Notably, the share of hospitals unsure of the source rose sharply from 1.0% in 2023 to 16.8% in 2024.

#### ML Model Evaluation

Among hospitals adopting ML in 2023, 62.6% reported evaluating model accuracy for all or most models, while only 45% assessed model bias (Figure S6 in Multimedia Appendix 1). These rates of model accuracy and bias evaluation increased to 70.7% and 56.9% in 2024. In 2024, 58.1% of the hospitals conducted postimplementation evaluation and monitoring.

### Regression Analysis Results 

#### Whether to Adopt Any ML

The IPW weighted regression estimates of the associations between ML adoption and hospital characteristics are reported in Table 2. Compared to nonfederal governmental hospitals, not-for-profit hospitals were 4.4 (95% CI 0.6-8.2) percentage points more likely to adopt any ML within EHR systems (*P*=.02), while for-profit hospitals were 8.5 (95% CI −15 to −2.1) percentage points (*P*=.009) less likely to adopt. Large hospitals were 15.2 (95% CI 9.4-21) percentage points more likely than small hospitals to adopt ML into EHRs (*P*<.001). Hospitals affiliated with a health system had a 26.8 (95% CI 22.4-31.3) percentage point higher likelihood of ML adoption (*P*<.001). In contrast, CAHs were 8.4 (95% CI –12.4 to –4.4) percentage points less likely to adopt ML (*P*<.001). Contracting with leading EHR vendors was associated with a 20.6 (95% CI 17.1-24) percentage points increase in ML adoption likelihood (*P*<.001). Hospitals located in metropolitan areas were 4.3 (95% CI 0.8-7.8) percentage points more likely to adopt ML technology compared to those in nonmetropolitan areas (*P*=.02).

**Table 2.** Associations between hospital characteristics and machine learning adoption^a^.

	Marginal effects	95% CI	*P* value
Organizational context			
Hospital type			
Nonfederal, governmental	Reference	—^b^	—
Not-for-profit	4.4^c^	0.6 to 8.2	.02
For profit	–8.5^d^	–14.9 to –2.1	.009
Hospital size			
Small, 0‐99 beds	Reference	—	—
Medium, 100‐399 beds	3.2	–0.7 to 7.2	.11
Large, 400 or more beds	15.2^e^	9.4 to 21.0	<.001
Critical access hospital	–8.4^e^	–12.4 to –4.4	<.001
Teaching status	–2.1	–12.2 to 7.9	.68
Environmental context			
Health system	26.8^e^	22.4 to 31.3	<.001
Leading EHR^f^^,^^g^	20.6^e^	17.1 to 24.0	<.001
Metropolitan^h^	4.3^c^	0.8 to 7.8	.02
Census division			
New England	Reference	—	—
Middle Atlantic	–2.7	–10.8 to 5.4	.51
East North Central	0.7	–6.9 to 8.2	.86
West North Central	6.8	–0.5 to 14.1	.07
South Atlantic	9.4^c^	2.0 to 16.8	.01
East South Central	2.8	–5.9 to 11.4	.53
West South Central	–1.8	–9.4 to 5.8	.65
Mountain	6.3	–1.2 to 13.8	.10
Pacific	–2.0	–10.1 to 6.1	.62
Year 2024	1.9^c^	0.04 to 3.7	.05

a Authors’ analysis of data from the 2022‐2023 American Hospital Association (AHA) Annual Survey linked with the 2023‐2024 AHA IT Supplement. The sample includes 4055 hospital-year observations. The table reports average marginal effects, which represent the change in the probability of any machine learning adoption associated with each hospital characteristic. Marginal effects are scaled by 100 and can be interpreted as percentage point changes. Estimates are derived from a weighted logistic regression model using inverse probability weights (derived from propensity scores) to account for IT Supplement nonresponse. SEs are clustered at the hospital level to account for within-hospital correlation over time.

b Not applicable

c *P*<.05.

d *P*<.01.

e *P*<.001.

f Leading EHR vendors are identified by market share.

g EHR: electronic health record.

h Metropolitan status is categorized based on 2023 Rural-Urban Continuum Codes: 1‐3 as metropolitan counties and 4‐9 as nonmetropolitan counties.

#### Type of ML Adoption

The multinomial logistic regression results in Table 3 illustrate the association between hospital characteristics and 4 types of ML adoption. The results of both clinical and operational ML adoption are overall consistent with the estimates on any ML adoption: not-for-profit, large size, health system affiliation, non-CAH, leading EHR contract, and metropolitan location were related to both ML adoption. Regarding other types of ML adoption, for-profit (−15.1; 95% CI −20 to −10.2; *P*<.001) and teaching (−6.2; 95% CI −11 to −1.4; *P*<.001) hospitals were less likely to adopt only clinical ML. Large size (−1; 95% CI −2 to −0.1; *P*=.03) and health system affiliation (−1.8; 95% CI −3.4 to −0.2; *P*=.03) were related to a lower likelihood of only operational ML adoption.

**Table 3.** Associations between hospital characteristics and types of machine learning adoption in electronic health records^a^.

	Marginal effects	95% CI	*P* value
Panel A: clinical ML^b^ only			
Organizational context			
Hospital type			
Nonfederal, governmental	Reference	—^i^	—
Not-for-profit	–1.2	–5.7 to 3.3	.60
For profit	–15.1^c^	–20.0 to –10.2	<.001
Hospital size			
Small, 0‐99 beds	Reference	—	—
Medium, 100‐399 beds	–0.1	–3.8 to 3.5	.95
Large, 400 or more beds	2.7	–3.3 to 8.7	.38
Critical access hospital	1.9	–2.0 to 5.9	.34
Teaching status	–6.2^d^	–11.0 to –1.4	.01
Environmental context			
Health system	2.3	–1.8 to 6.5	.28
Leading EHR^e^^,^^f^	–0.4	–3.8 to 2.9	.80
Metropolitan^g^	0	–3.4 to 3.4	.98
Census division			
New England	Reference	—	—
Middle Atlantic	–7.8	–16.6 to 1.0	.08
East North Central	–16.8^c^	–24.6 to –9.0	<.001
West North Central	–8.4^d^	–16.7 to –0.1	.05
South Atlantic	–15.5^c^	–23.5 to –7.5	<.001
East South Central	1.2	–8.7 to 11.1	.82
West South Central	–13.7^c^	–21.9 to –5.6	<.001
Mountain	–12.5^h^	–21.0 to –4.1	.004
Pacific	–17.9^c^	–26.1 to –9.8	<.001
Year 2024	–8.8^c^	–10.9 to –6.7	<.001
Panel B: operational ML only			
Organizational context			
Hospital type			
Nonfederal, governmental	Reference	—	—
Not-for-profit	0.4	–0.7 to 1.6	.45
For profit	–0.4	–1.9 to 1.1	.59
Hospital size			
Small, 0‐99 beds	Reference	—	—
Medium, 100‐399 beds	0.3	–0.7 to 1.4	.52
Large, 400 or more beds	–1.0^d^	–2.0 to –0.1	.03
Critical access hospital	–0.5	–1.7 to 0.7	.40
Teaching status	1.6	–1.7 to 4.9	.34
Environmental context			
Health system	–1.8^d^	–3.4 to –0.2	.03
Leading EHR	0.6	–0.1 to 1.3	.11
Metropolitan	–0.2	–1.3 to 0.9	.72
Census division			
New England	Reference	—	—
Middle Atlantic	–0.7	–2.0 to 0.6	.31
East North Central	1.6	–0.1 to 3.3	.06
West North Central	0.5	–1.4 to 2.3	.61
South Atlantic	2.0^d^	0.2 to 3.8	.03
East South Central	0.1	–1.8 to 1.9	.94
West South Central	0.2	–1.6 to 2.0	.80
Mountain	0	–1.6 to 1.6	.97
Pacific	–0.9	–2.1 to 0.4	.17
Year 2024	1.6^c^	0.9 to 2.3	<.001
Panel C: both types			
Organizational context			
Hospital type			
Nonfederal, governmental	Reference	—	—
Not-for-profit	6.0^d^	0.8 to 11.1	.02
For profit	7.6^d^	0.4 to 14.8	.04
Hospital size			
Small, 0‐99 beds	Reference	—	—
Medium, 100‐399 beds	2.8	–1.7 to 7.3	.22
Large, 400 or more beds	13.5^c^	6.7 to 20.2	<.001
Critical access hospital	–9.9^c^	–14.8 to –5.0	<.001
Teaching status	3.2	–5.7 to 12.0	.48
Environmental context			
Health system	26.5^c^	21.7 to 31.3	<.001
Leading EHR	20.5^c^	16.6 to 24.3	<.001
Metropolitan	4.6^d^	0.3 to 8.9	.03
Census division			
New England	Reference	—	—
Middle Atlantic	5.9	–3.8 to 15.7	.23
East North Central	16.2^c^	6.9 to 25.5	<.001
West North Central	14.7^h^	5.4 to 24.0	.002
South Atlantic	23.0^c^	13.7 to 32.3	<.001
East South Central	1.7	–9.0 to 12.4	.75
West South Central	11.9^d^	2.3 to 21.5	.02
Mountain	19.0^c^	9.0 to 28.9	<.001
Pacific	17.3^c^	7.4 to 27.1	<.001
Year 2024	8.8^c^	6.5 to 11.1	<.001

a Authors’ analysis of data from the 2022‐2023 American Hospital Association (AHA) Annual Survey linked with the 2023‐2024 AHA Information Technology (IT) Supplement. The sample includes 4055 hospital-year observations. The table presents average marginal effects (MEs) from a single multinomial logistic regression, where the outcome is a 4-category measure of ML adoption type: clinical only (Panel A), operational only (Panel B), both (Panel C), and no ML adoption (the base outcome). Each ME represents the change in the probability of being in a specific category associated with a hospital characteristic. The model is weighted using inverse probability weights (derived from propensity scores) to account for the IT Supplement nonresponse. MEs are scaled by 100 and can be interpreted as percentage point changes. SEs are clustered at the hospital level to account for within-hospital correlation over time.

b ML: machine learning.

c *P*<.001.

d *P*<.05.

e Leading EHR vendors are identified by market share.

f EHR: electronic health record.

g Metropolitan status is categorized based on the 2023 Rural-Urban Continuum Codes: 1‐3 as metropolitan counties and 4‐9 as nonmetropolitan counties.

h *P*<.01.

i Not applicable.

#### Individual ML Functions

We further explored the relationship between hospital features and 6 individual ML functions in Tables S2 and S3 in Multimedia Appendix 1. Overall, the patterns were consistent with the main findings in Tables 2 and 3, but some exceptions emerged. Nonprofit hospitals showed a stronger emphasis on clinical applications, including predicting health risks for inpatients (7.5; 95% CI 3.6-11.4; *P*<.001) and outpatients (7.7; 95% CI 3.4-12; *P*<.001). In contrast, for-profit hospitals (26.4; 95% CI 19.3-33.6; *P*<.001) and large hospitals (11.6; 95% CI 5.3-18; *P*<.001) were more likely to adopt scheduling-related ML functions. To enable clearer review and a comparison of evidence across various sets of outcomes, we provided a summary overview in Table 4.

**Table 4.** Summary of all regression results.

	Overall	Types of adoption			Adoption of individual function					
		Clinical only	Operational only	Both types	Predict inpatient risk	Predict outpatient risk	Monitor health	Recommend treatments	Billing	Scheduling
Organizational context										
Hospital type (reference: nonfederal, governmental)										
Not-for-profit	+^a^	NS^b^	NS	+	+	+	NS	NS	NS	NS
For profit	–^c^	–	NS	+	NS	–	–	–	NS	+
Hospital size (reference: small, 0-99 beds)										
Medium, 100‐399 beds	NS	NS	NS	NS	NS	NS	NS	NS	NS	+
Large, 400 or more beds	+	NS	–	+	+	+	NS	NS	+	+
Critical access hospital	–	NS	NS	–	–	–	NS	NS	NS	–
Teaching status	NS	–	NS	NS	NS	NS	NS	NS	NS	NS
Environmental context										
Health system	+	NS	NS	+	+	+	+	+	+	+
Leading EHR^d^^,^^e^	+	NS	NS	+	+	+	+	+	+	+
Metropolitan^f^	+	NS	NS	+	NS	NS	+	+	NS	NS

a + means a significant positive association at the .05 level.

b NS means no significant association.

c – means a significant negative association at the .05 level.

d Leading EHR vendors are identified by market share.

e EHR: electronic health record.

f Metropolitan status is categorized based on 2023 Rural-Urban Continuum Codes: 1‐3 as metropolitan counties and 4‐9 as nonmetropolitan counties.

#### Additional Analysis Results 

To check the robustness of our findings, we estimated models without IPWs and compared them with our main estimates (Tables S4 and S5 in Multimedia Appendix 1). We found both weighted and nonweighted estimates converge. The estimates are consistent with prior research on hospital AI adoption for workforce optimization [28,29], lending credibility to our findings.

 We also conducted the subgroup analysis to detect heterogeneity patterns in different hospital sizes (Table S6 in Multimedia Appendix 1). Health system affiliation and leading EHR vendors significantly increased the probability of ML adoption, and both effects are larger in small hospitals than in medium hospitals.

## Discussion

### Principal Results

This study provides timely, national-level evidence on ML adoption within hospital EHR systems, revealing substantial variation in specific functions, model evaluation practices, and hospital characteristics. Consistent with prior research on overall ML adoption, organizational and environmental factors such as hospital type, size, CAH status, health system membership, and metropolitan location remain key determinants of adoption decisions, supporting the organizational and environmental contexts in the TOE framework. However, our findings extend beyond existing studies by identifying marked heterogeneity in the adoption of individual ML domains and functions, highlighting that hospitals differ not only in whether they adopt ML but also in which types of ML applications they prioritize. Such heterogeneity aligns well with the technological context in the TOE framework, suggesting that ML adoption may be influenced by each hospital’s assessment of the relative benefits and costs. Understanding this heterogeneity is essential for designing further policies and implementation strategies that promote equitable and effective ML diffusion across diverse health care settings.

### Resource-Based Explanation

Within the TOE framework, the organizational and environmental contexts offer a resource-based explanation for our findings, suggesting that both internal and external resources facilitate or restrict hospital technology adoption. Prior literature has identified such resources as key determinants of EHR adoption [40,48]. Our findings similarly suggest that hospitals with characteristics commonly associated with greater organizational capacity (eg, larger size, urban location, and system affiliation) were more likely to report ML adoption. Although we did not directly document the decision-making process or measure organizational resources, these characteristics may serve as proxies for financial resources, human capital, IT capabilities, and leadership support. Large, urban, and system-affiliated hospitals typically possess the financial flexibility to purchase new add-on functions from EHR vendors or develop custom ML algorithms tailored to their specific needs. Their established IT infrastructure facilitates efficient ML adoption within EHRs, and their resource-rich environments often have sufficient staffing and training programs to support implementation. Executive leadership and strategic vision also play a critical role in advancing ML adoption [49].

  Further, our findings suggest that the unequal distribution of health care resources acts as a significant barrier to ML adoption for hospitals in rural and underserved areas [50-52] and that external resource support could facilitate ML adoption in such hospitals. Our subgroup analysis findings showed that health system affiliation and leading EHR contract had stronger effects on small hospitals, partly due to the greater availability of financial and technical support.

### Heterogeneous ML Adoption

Although most hospitals tend to adopt both clinical and operational ML functions simultaneously, specific hospitals select particular ML applications in alignment with their technical needs, as the TOE framework suggests. Most notably, for-profit hospitals demonstrated a strong interest in adopting ML regarding scheduling functions, which may be explained by staffing structures and institutional objectives. They more frequently experience staffing shortages [53] and higher nursing turnover rates [54]. In this context, ML-enabled scheduling tools offer a practical solution for maintaining service quality at a lower cost by automating front-office functions and reducing reliance on staff.

These findings suggest that hospitals vary in their perceptions, motivations, and priorities when adopting ML technologies, which are often shaped by their resources and strategic goals. This variability highlights the need for qualitative research and targeted surveys on the motives, facilitators, and barriers to implementing emerging ML technologies among health care providers and health system or hospital managers. For instance, researchers may explore how hospital managers perceive and evaluate specific ML technology, enablers and challenges during implementation, and whether ML models meet their expectations.

### Limited Model Evaluation

Our findings highlight a notable gap in current hospital AI practice: limited evaluation of models using local hospital or health system data [55-57]. Given that ML performance is highly dependent on the distribution and quality of the training data in specific contexts, its real-world effectiveness and validity in dynamic health care environments are often unclear and may degrade for specific patients. Moreover, if the training data contain inherent biases, it may perpetuate or even exacerbate existing systemic disparities among vulnerable populations [55-57].

Researchers in venues such as the Machine Learning for Healthcare conference and the Conference on Human Factors in Computing Systems have emphasized practical frameworks for evaluating ML quality in care delivery, including real-world effectiveness testing, model actionability, model lifecycle management, and performance tracking across sites and settings [58-60]. To mitigate risk and support safe integration, developers and scholars recommend tailoring evaluation to the clinical context, engaging clinicians and patients, increasing transparency, labeling bias across patient groups, and fostering a supportive organizational culture [61-63]. This gap also underscores the need for governmental and industrial initiatives and regulations to promote the safe, effective, and trustworthy use of ML technologies [64]. Although nascent [65-67], these efforts could provide critical oversight, standards, and incentives that help minimize risks and encourage responsible implementation.

### Policy or Practice Suggestion

Multi-level policy interventions are necessary to ensure equitable ML adoption and bridge the digital divide across health care settings. The federal government may introduce an initiative similar to the Health Information Technology for Economic and Clinical Health Act of 2009, which significantly accelerated the adoption of meaningful EHR use among rural and small hospitals [68,69]. These interventions could include expanding IT broadband infrastructure in underserved areas or providing direct financial incentives to support ML adoption. Meanwhile, health systems and EHR vendors should consider providing additional financial and technical support specifically for rural and small hospitals. Especially, EHR vendors should prioritize integrating foundational ML functions within the existing EHR systems to reduce the technical barriers of meaningful ML usage. Also, offering discounted or complimentary updates for ML features may be a valuable strategy to support hospitals with limited resources.

### Limitation

Our study has several limitations. First, our findings should be interpreted as correlational rather than causal, due to unobserved factors. Second, our analysis relies on secondary data from the AHA, which may be subject to self-report bias. Although our design cannot fully address these biases, the AHA data are widely used in health services research as reliable data sources, and they provide unique and detailed information on hospitals’ adoption of ML in EHR unavailable elsewhere. Future studies could validate the AHA data through primary data collection or cross-validation with objective measures. Third, our measures of ML adoption are self-reported by hospital managers, which may be subject to recall error, social-desirability bias, or heterogeneous interpretations of ML functionality. As a result, hospitals may overreport or misreport their level of ML adoption. In addition, we lack several granular contextual variables such as vendor-specific implementation details, interoperability maturity, IT workforce perspectives, and organizational culture, which provide necessary scenarios to understand ML implementation in practice. To address these gaps, future researchers could leverage qualitative and mixed methods designs (eg, interview or focus groups with clinicians, hospital managers, IT staff, and vendor representatives) to capture nuanced adoption processes, identify facilitators and barriers, and document practical implementation strategies. Fourth, the AHA IT Supplement Survey began collecting ML implementation in 2023, which limited our ability to capture early efforts in ML adoption within EHR. Finally, 44.7% of the AHA hospitals did not complete the IT Supplement Survey, raising concerns about potential nonresponse bias. Although we applied inverse propensity score weighting to balance observed characteristics between respondents and nonrespondents, which could strengthen our findings, the nonresponse bias remains if unobserved characteristics are correlated with both survey response and ML adoption.

### Conclusion

In this retrospective study of US hospitals from 2022 to 2024, we observed a high rate of ML adoption within EHR systems and considerable variation across specific ML functions. We also identified several hospital characteristics associated with ML adoption within EHRs, including ownership, hospital size, health system affiliation, CAH status, and metropolitan location. Given that resource availability significantly influences a hospital’s capacity to implement ML technologies within EHRs, our findings highlight the need for policy interventions that provide financial and technical support. Such efforts are essential to ensure that resource-constrained hospitals can adopt emerging health IT innovations and prevent the widening of digital and health disparities across the health care system.

To ensure accuracy, fairness, and safety, health care administrators and policymakers must prioritize not only the adoption of ML technologies but also the ongoing monitoring and evaluation of these tools. Equitable access to these technologies must be a key focus, with targeted support for hospitals facing barriers to adoption. By fostering inclusive and transparent approaches to ML adoption, we can maximize its potential to improve care delivery, reduce disparities, and enhance health care outcomes for all patients.

## Supplementary material

10.2196/76126 Multimedia Appendix 1 Additional methodological details, descriptive analyses, and robustness checks that complement the main text.

## Abbreviations

AHA: American Hospital Association
AI: artificial intelligence
CAH: critical access hospital
EHR: electronic health record
IPW: inverse probability weight
ML: machine learning
STROBE: Strengthening the Reporting of Observational Studies in Epidemiology
TOE: Technology-Organization-Environment
WRDS: Wharton Research Data Services

## References

[1] Matheny M, Israni ST, Ahmed M, Whicher D (2019). Artificial Intelligence in Health Care: The Hope, the Hype, the Promise, the Peril.

[2] Alowais SA, Alghamdi SS, Alsuhebany N (2023). Revolutionizing healthcare: the role of artificial intelligence in clinical practice. BMC Med Educ.

[3] Ahsan MM, Luna SA, Siddique Z (2022). Machine-learning-based disease diagnosis: a comprehensive review. Healthcare (Basel).

[4] Nelson KM, Chang ET, Zulman DM, Rubenstein LV, Kirkland FD, Fihn SD (2019). Using predictive analytics to guide patient care and research in a national health system. J Gen Intern Med.

[5] Donzé J, Aujesky D, Williams D, Schnipper JL (2013). Potentially avoidable 30-day hospital readmissions in medical patients: derivation and validation of a prediction model. JAMA Intern Med.

[6] Subramanian M, Wojtusciszyn A, Favre L (2020). Precision medicine in the era of artificial intelligence: implications in chronic disease management. J Transl Med.

[7] Haug CJ, Drazen JM (2023). Artificial intelligence and machine learning in clinical medicine. N Engl J Med.

[8] Pianykh OS, Guitron S, Parke D (2020). Improving healthcare operations management with machine learning. Nat Mach Intell.

[9] Pachamanova D, Tilson V, Dwyer-Matzky K (2022). Case article—machine learning, ethics, and change management: a data-driven approach to improving hospital observation unit operations. INFORMS Trans Educ.

[10] Bhagat SV, Kanyal D (2024). Navigating the future: the transformative impact of artificial intelligence on hospital management—a comprehensive review. Cureus.

[11] Maleki Varnosfaderani S, Forouzanfar M (2024). The role of AI in hospitals and clinics: transforming healthcare in the 21st century. Bioengineering (Basel).

[12] (2021). AI survey: health care organizations continue to adopt artificial intelligence to help achieve better, more equitable and affordable patient outcomes. UnitedHealth Group.

[13] (2024). AI and the future of healthcare: applications, compliance, and opportunities for artificial intelligence in healthcare and pharma. Berkeley Research Group.

[14] Sahni N, Stein G, McKinsey O, Zemmel R, Cutler DM (2023). The Potential Impact of Artificial Intelligence on Healthcare Spending.

[15] Ryu AJ, Ayanian S, Qian R (2023). A clinician’s guide to running custom machine-learning models in an electronic health record environment. Mayo Clin Proc.

[16] Kawamoto K, Finkelstein J, Del Fiol G (2023). Implementing machine learning in the electronic health record: checklist of essential considerations. Mayo Clin Proc.

[17] Capoot A (2024). Epic systems is building more than 100 new AI features for doctors and patients here’s what’s coming. CNBC.

[18] Wen A, Fu S, Moon S (2019). Desiderata for delivering NLP to accelerate healthcare AI advancement and a mayo clinic NLP-as-a-service implementation. NPJ Digit Med.

[19] Tsai ML, Chen KF, Chen PC (2025). Harnessing electronic health records and artificial intelligence for enhanced cardiovascular risk prediction: a comprehensive review. J Am Heart Assoc.

[20] Hernandez-Boussard T, Monda KL, Crespo BC, Riskin D (2019). Real world evidence in cardiovascular medicine: ensuring data validity in electronic health record-based studies. J Am Med Inform Assoc.

[21] Budd J (2023). Burnout related to electronic health record use in primary care. J Prim Care Community Health.

[22] Adler-Milstein J, Zhao W, Willard-Grace R, Knox M, Grumbach K (2020). Electronic health records and burnout: time spent on the electronic health record after hours and message volume associated with exhaustion but not with cynicism among primary care clinicians. J Am Med Inform Assoc.

[23] Chaudoir SR, Dugan AG, Barr CH (2013). Measuring factors affecting implementation of health innovations: a systematic review of structural, organizational, provider, patient, and innovation level measures. Implementation Sci.

[24] Seneviratne MG, Shah NH, Chu L (2020). Bridging the implementation gap of machine learning in healthcare. BMJ Innov.

[25] (2024). Principles for augmented intelligence development, deployment, and use. American Medical Association.

[26] Amarasingham R, Patzer RE, Huesch M, Nguyen NQ, Xie B (2014). Implementing electronic health care predictive analytics: considerations and challenges. Health Aff (Millwood).

[27] Hua D, Petrina N, Young N, Cho JG, Poon SK (2024). Understanding the factors influencing acceptability of AI in medical imaging domains among healthcare professionals: a scoping review. Artif Intell Med.

[28] Bin Abdul Baten R (2024). How are US hospitals adopting artificial intelligence? Early evidence from 2022. Health Aff Sch.

[29] Chen J, Yan AS (2025). Hospital artificial intelligence/machine learning adoption by neighborhood deprivation. Med Care.

[30] Nong P, Adler-Milstein J, Apathy NC, Holmgren AJ, Everson J (2025). Current use and evaluation of artificial intelligence and predictive models in US hospitals. Health Aff (Millwood).

[31] (2022). Clinical decision support software: guidance for industry and Food and Drug Administration staff. US Food & Drug Administration.

[32] Knight DRT, Aakre CA, Anstine CV (2023). Artificial intelligence for patient scheduling in the real-world health care setting: a metanarrative review. Health Policy Technol.

[33] Talwar A, Lopez-Olivo MA, Huang Y, Ying L, Aparasu RR (2023). Performance of advanced machine learning algorithms overlogistic regression in predicting hospital readmissions: a meta-analysis. Explor Res Clin Soc Pharm.

[34] Baker J (2011). Information Systems Theory: Explaining and Predicting Our Digital Society.

[35] Tornatzky LG, Fleischer M (1990). Processes of Technological Innovation.

[36] Ramdani B, Duan B, Berrou I (2020). Exploring the determinants of mobile health adoption by hospitals in China: empirical study. JMIR Med Inform.

[37] Beier M, Früh S (2020). Technological, organizational, and environmental factors influencing social media adoption by hospitals in Switzerland: cross-sectional study. J Med Internet Res.

[38] Ahmadi H, Nilashi M, Ibrahim O (2015). Organizational decision to adopt hospital information system: an empirical investigation in the case of Malaysian public hospitals. Int J Med Inform.

[39] (2025). AHA Data.

[40] Kruse CS, Kothman K, Anerobi K, Abanaka L (2016). Adoption factors of the electronic health record: a systematic review. JMIR Med Inform.

[41] Chang W, Owusu-Mensah P, Everson J, Richwine C (2025). Hospital trends in the use, evaluation, and governance of predictive AI, 2023-2024. Assistant Secretary for Technology Policy.

[42] Norton EC, Dowd BE, Garrido MM, Maciejewski ML (2024). Requiem for odds ratios. Health Serv Res.

[43] Norton EC, Dowd BE, Maciejewski ML (2019). Marginal effects-quantifying the effect of changes in risk factors in logistic regression models. JAMA.

[44] (2025). Stata Statistical Software: release 18. StataCorp.

[45] Funk MJ, Westreich D, Wiesen C, Stürmer T, Brookhart MA, Davidian M (2011). Doubly robust estimation of causal effects. Am J Epidemiol.

[46] Richwine C, Meklir S (2024). Hospitals’ collection and use of data to address social needs and social determinants of health. Health Serv Res.

[47] (2017). Protection of human subjects, 45 CFR § 46. Code of Federal Regulations.

[48] Houser SH, Au D, Weech-Maldonado R (2011). The impact of geography on hospital electronic health records implementation in Alabama: implications for meaningful use. Appl Clin Inform.

[49] Pumplun L, Fecho M, Wahl N, Peters F, Buxmann P (2021). Adoption of machine learning systems for medical diagnostics in clinics: qualitative interview study. J Med Internet Res.

[50] Hassan M, Kushniruk A, Borycki E (2024). Barriers to and facilitators of artificial intelligence adoption in health care: scoping review. JMIR Hum Factors.

[51] Eltawil FA, Atalla M, Boulos E, Amirabadi A, Tyrrell PN (2023). Analyzing barriers and enablers for the acceptance of artificial intelligence innovations into radiology practice: a scoping review. Tomography.

[52] Wenderott K, Krups J, Weigl M, Wooldridge AR (2025). Facilitators and barriers to implementing AI in routine medical imaging: systematic review and qualitative analysis. J Med Internet Res.

[53] (2022). Care crisis: how low staffing contributes to patient care failure at HCA hospitals. HCACareCrisis.

[54] Winter V, Schreyögg J, Thiel A (2020). Hospital staff shortages: environmental and organizational determinants and implications for patient satisfaction. Health Policy.

[55] Chin MH, Afsar-Manesh N, Bierman AS (2023). Guiding principles to address the impact of algorithm bias on racial and ethnic disparities in health and health care. JAMA Netw Open.

[56] Gervasi SS, Chen IY, Smith-McLallen A, Sontag D, Obermeyer Z, Vennera M (2022). The potential for bias in machine learning and opportunities for health insurers to address it: article examines the potential for bias in machine learning and opportunities for health insurers to address it. Health Aff.

[57] Obermeyer Z, Powers B, Vogeli C, Mullainathan S (2019). Dissecting racial bias in an algorithm used to manage the health of populations. Science.

[58] Zając HD, Ribeiro JMN, Ingala S (2024). “It depends”: configuring AI to improve clinical usefulness across contexts.

[59] Ehrmann DE, Joshi S, Goodfellow SD, Mazwi ML, Eytan D (2023). Making machine learning matter to clinicians: model actionability in medical decision-making. NPJ Digit Med.

[60] Bedoya AD, Economou-Zavlanos NJ, Goldstein BA (2022). A framework for the oversight and local deployment of safe and high-quality prediction models. J Am Med Inform Assoc.

[61] Nichol AA, Sankar PL, Halley MC, Federico CA, Cho MK (2023). Developer perspectives on potential harms of machine learning predictive analytics in health care: qualitative analysis. J Med Internet Res.

[62] Chang T, Sjoding MW, Wiens J (2022). Disparate censorship & undertesting: a source of label bias in clinical machine learning. Proc Mach Learn Res.

[63] McCradden MD, Kirsch RE (2023). Patient wisdom should be incorporated into health AI to avoid algorithmic paternalism. Nat Med.

[64] Matheny ME, Goldsack JC, Saria S (2025). Artificial intelligence in health and health care: priorities for action. Health Aff (Millwood).

[65] (2025). Guidance on the responsible use of AI in healthcare (RUAIH). Joint Commission and Coalition for Health AI.

[66] (2023). Artificial intelligence risk management framework (AI RMF 1.0). National Institute of Standards and Technology.

[67] Augenstein J, Seigel R, Shashoua M (2025). Manatt health: health AI policy tracker. Manatt.

[68] Adler-Milstein J, Jha AK (2017). HITECH Act drove large gains in hospital electronic health record adoption. Health Aff (Millwood).

[69] Everson J, Rubin JC, Friedman CP (2020). Reconsidering hospital EHR adoption at the dawn of HITECH: implications of the reported 9% adoption of a “basic” EHR. J Am Med Inform Assoc.

[70] AHA data product advisory. AHA Data.

[71] Wharton Research Data Services.

