# Supplementary material for: Adoption of Machine Learning in US Hospital Electronic Health Record Systems: Retrospective Observational Study
Source: J Med Internet Res. 2025 Dec 9;27:e76126. doi: 10.2196/76126 (PMC12688049; doi:10.2196/76126)
Supplement: Multimedia Appendix 1 [file jmir-v27-e76126-s001.docx]

## Appendix

### List of Tables and Figures

Appendix Figure S1. Sample Flow Chart

Appendix Figure S2. Trends in ML Adoption in EHR System by Year

Appendix Figure S3. Geographic Distribution of Hospital Machine Learning Adoption by County

Appendix Figure S4. Trends in Specific Machine Learning Function Adoption in Electronic Health Records by Year

Appendix Figure S5. Trends in Developer Sources for Hospital Machine Learning in Electronic Health Records by Year

Appendix Figure S6. Trends in Hospitals’ Evaluation of Machine Learning Models by Year

Appendix Table S1. Unweighted and Weighted Hospital Characteristics (%) by IT Supplement Response Status

Appendix Table S2. Associations Between Hospital Characteristics and Adoption of Specific Clinical Machine Learning Functions

Appendix Table S3. Associations Between Hospital Characteristics and Adoption of Specific Operational Machine Learning Functions

Appendix Table S4. Sensitivity Analysis of Associations Between Hospital Characteristics and Machine Learning Adoption without Applying Inverse Probability Weight

Appendix Table S5. Sensitivity Analysis of Associations Between Hospital Characteristics and Types of Machine Learning Adoption in HER without Applying Inverse Probability Weight

Appendix Table S6. Associations Between Hospital Characteristics and Machine Learning Adoption, Separately for Small and Medium Hospitals

| Appendix Figure S1. Sample Flow Chart |
| --- |
| 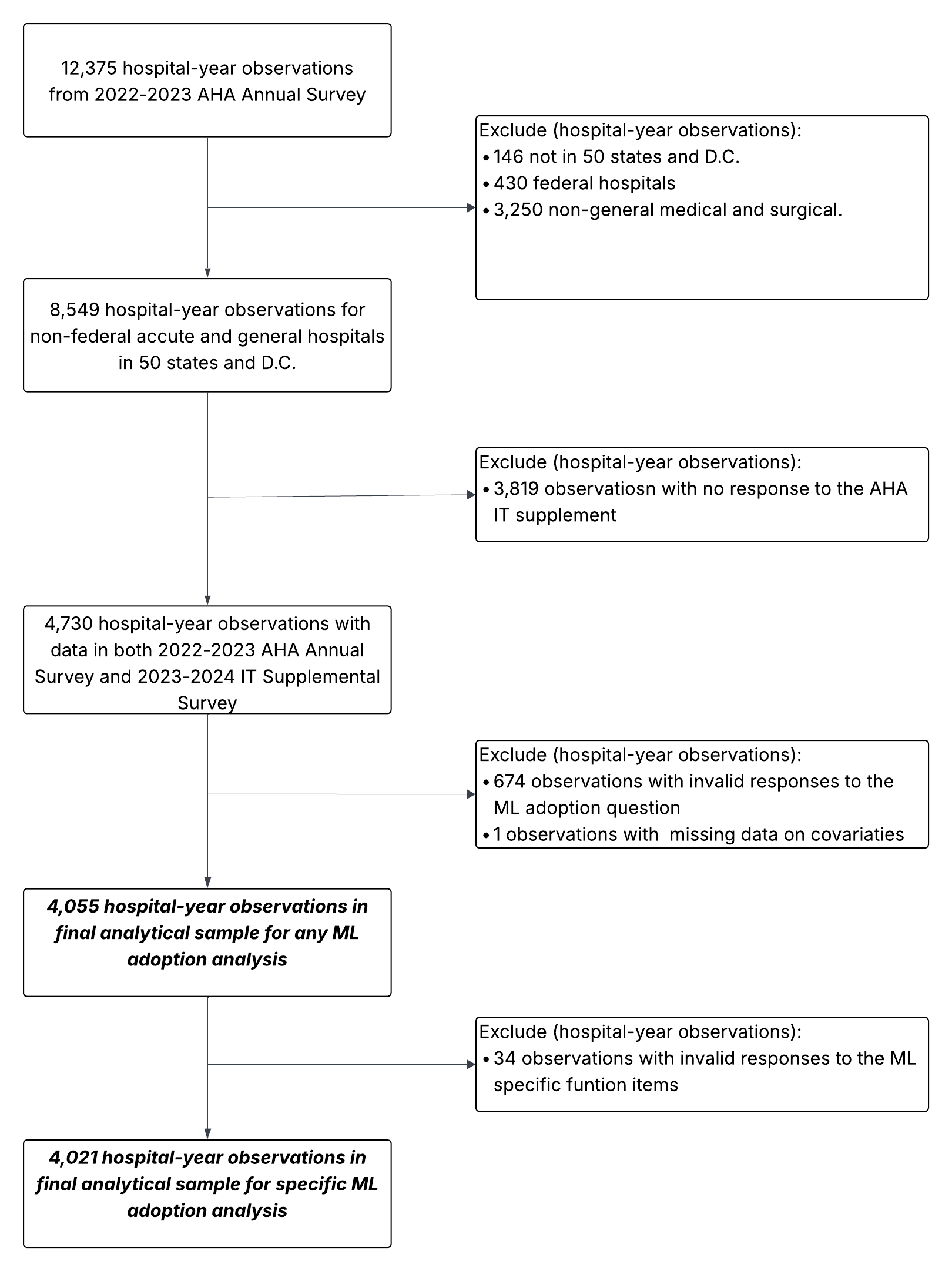 |
|  |

| **Appendix Figure S2. Trends in ML Adoption in EHR System by Year** |
| --- |
| 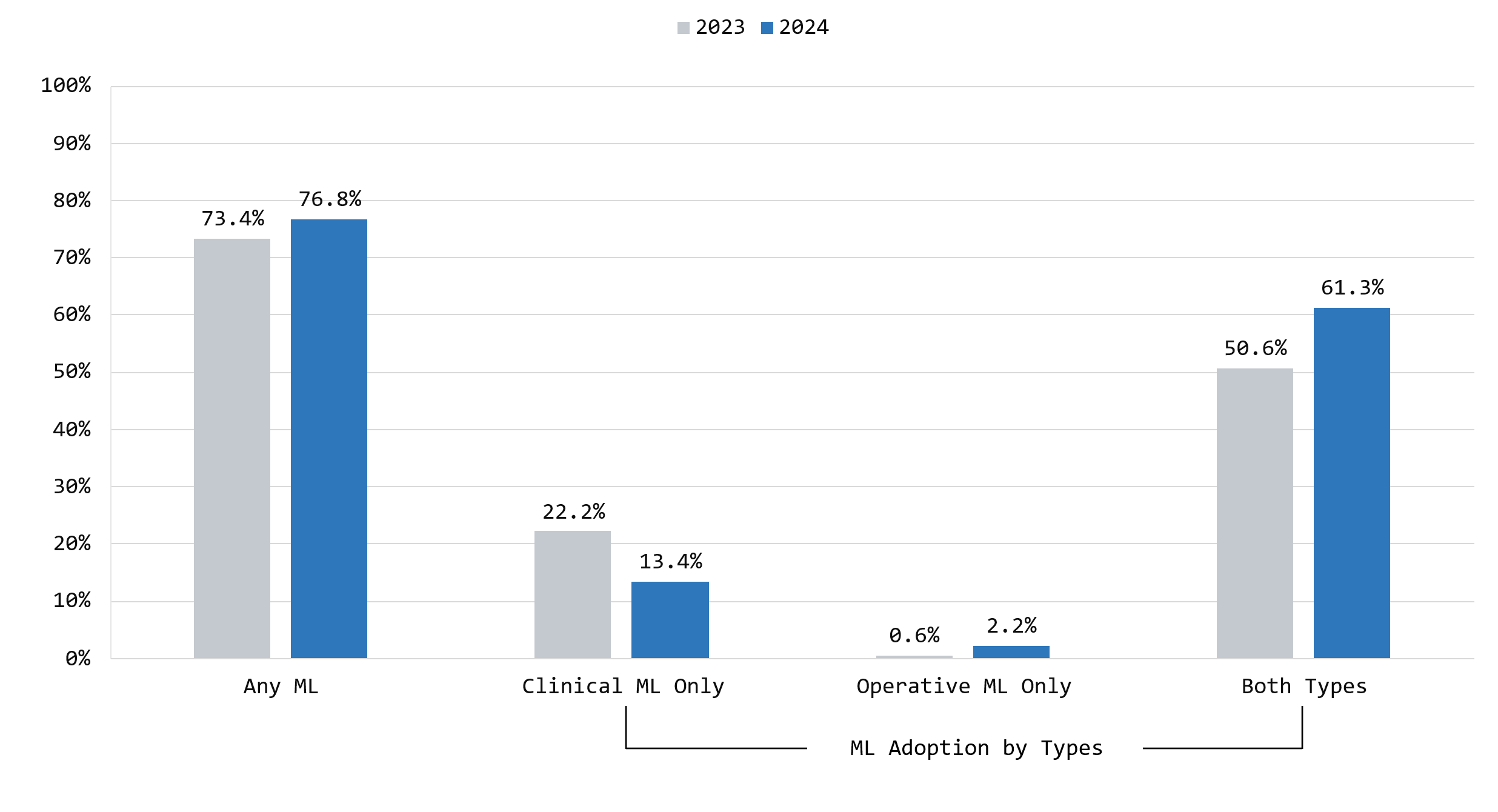 |
| Notes: Data are from the 2023–2024 American Hospital Association Information Technology (IT) Supplement survey. Percentages are weighted by inverse probability weight (derived from propensity scores) to account for IT Supplement nonresponse. Abbreviations: ML, Machine Learning. |

| **Appendix Figure S3. Geographic Distribution of Hospital Machine Learning Adoption by County** |
| --- |
| 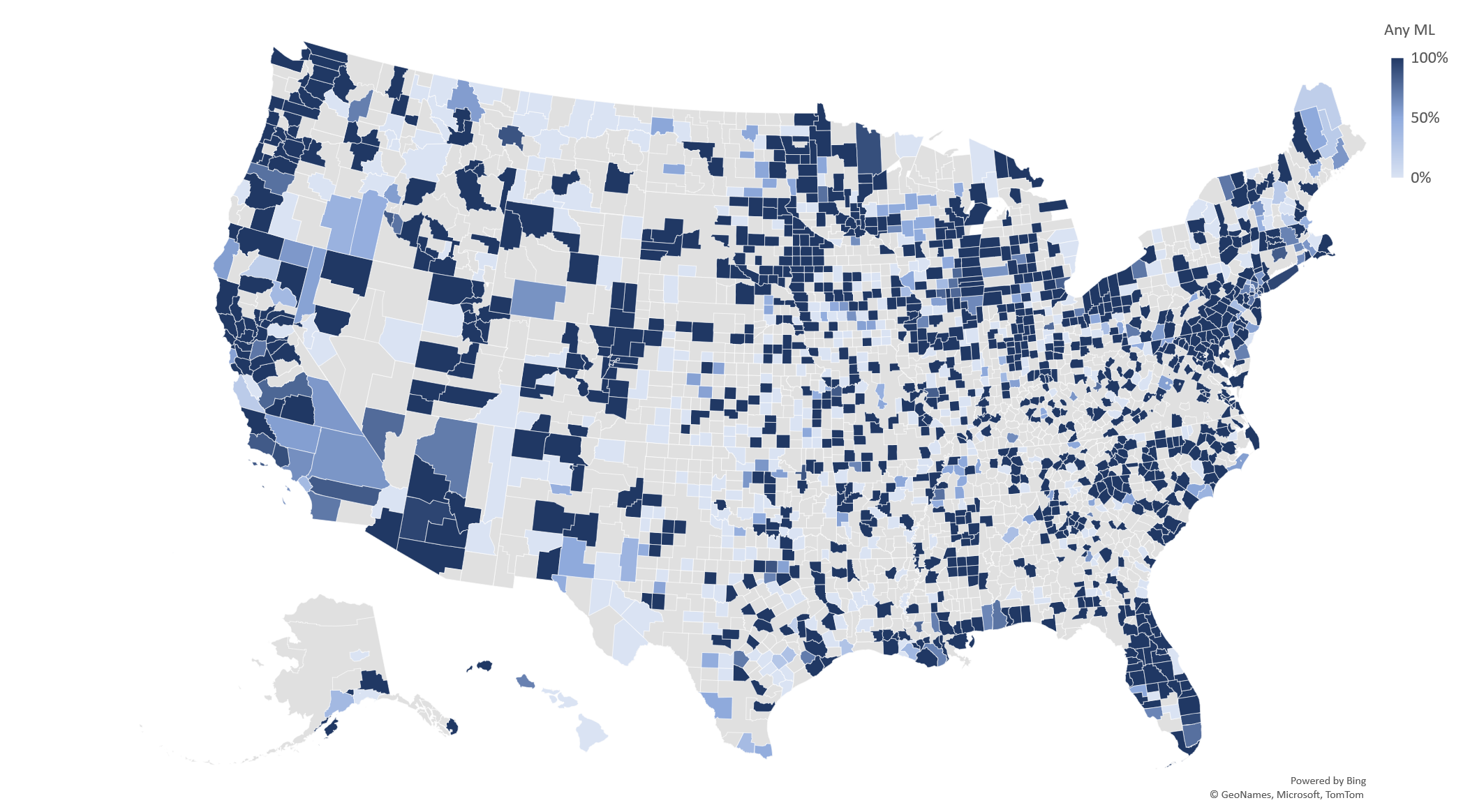 |
| Notes: Authors’ analysis of data are from the 2023–2024 American Hospital Association Information Technology (IT) Supplement survey. This map illustrates the county-level percentage of hospitals that have adopted any machine learning (ML) technology, pooling data from the 2023 and 2024 IT Supplement surveys. Adoption rates are weighted using inverse probability weights to account for survey nonresponse and ensure national representativeness. Counties with no available data are shaded in grey. The color intensity corresponds to the percentage of hospitals with ML adoption, as indicated by the legend. |

| **Appendix Figure S4. Trends in Specific Machine Learning Function Adoption in Electronic Health Records by Year** |
| --- |
| 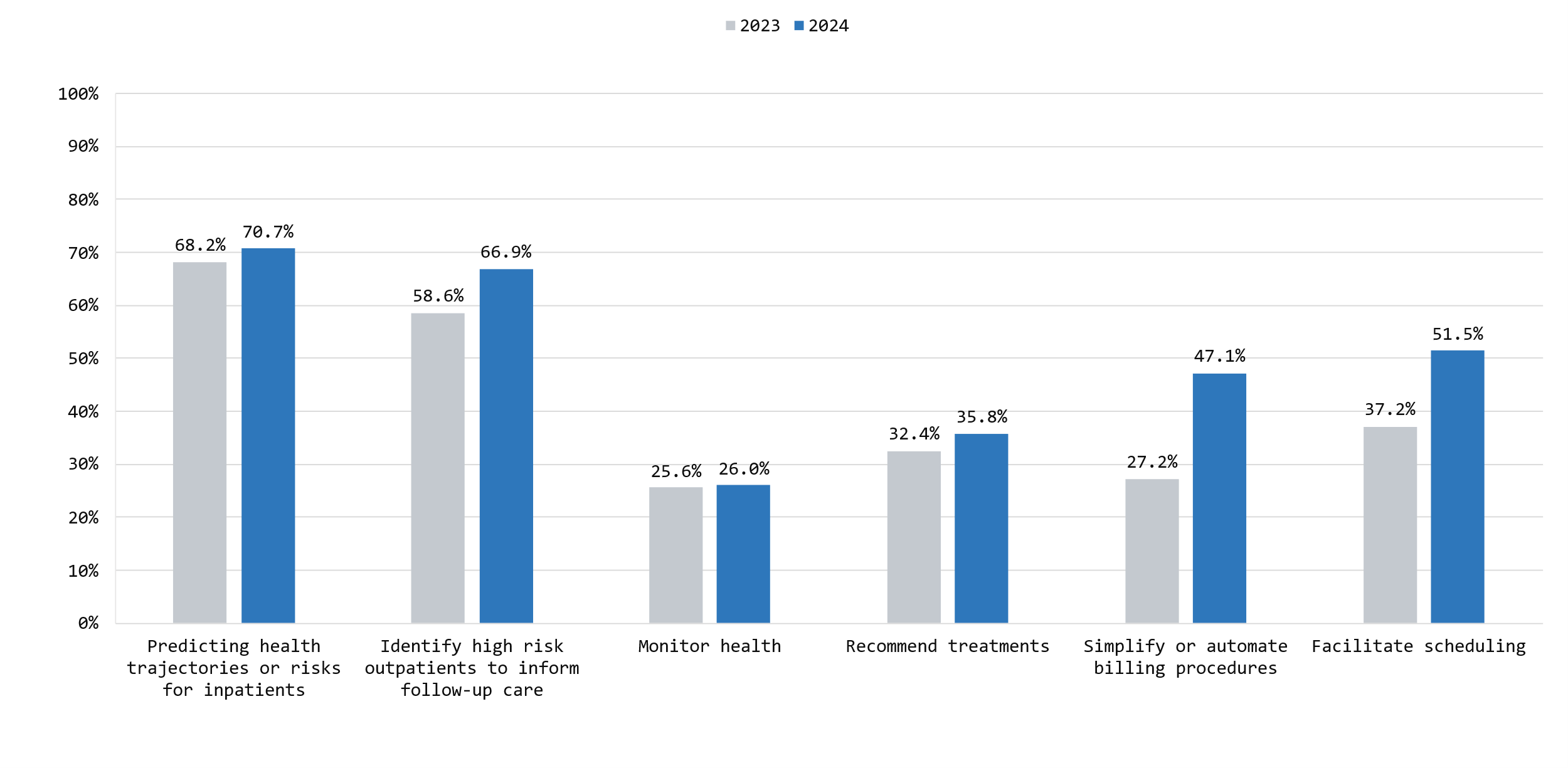 |
| Notes: Data are from the 2023–2024 American Hospital Association Information Technology (IT) Supplement survey. Percentages are weighted by inverse probability weight (derived from propensity scores) to account for IT Supplement nonresponse. |

| **Appendix Figure S5. Trends in Developer Sources for Hospital Machine Learning in Electronic Health Records by Year** |
| --- |
| 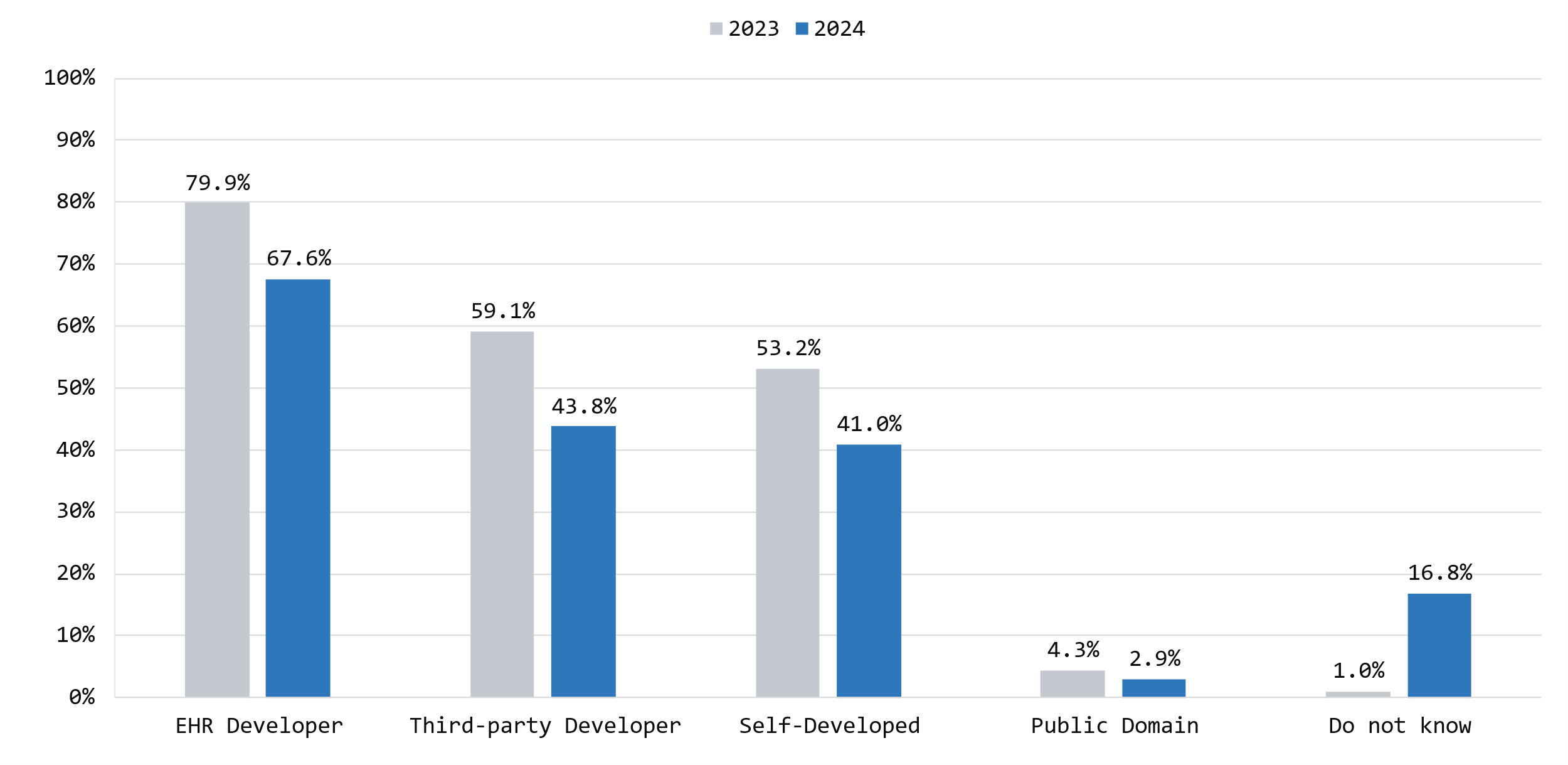 |
| Notes: Data are from the 2023–2024 American Hospital Association Information Technology (IT) Supplement survey. The figure shows the percentage of machine learning (ML) developer sources among ML-adopting hospitals. Hospitals could report using tools from multiple sources. Percentages are weighted by inverse probability weight (derived from propensity scores) to account for IT Supplement nonresponse. Abbreviations: EHR, Electronic Health Record. |

| **Appendix Figure S6. Trends in Hospitals’ Evaluation of Machine Learning Models by Year** |
| --- |
| 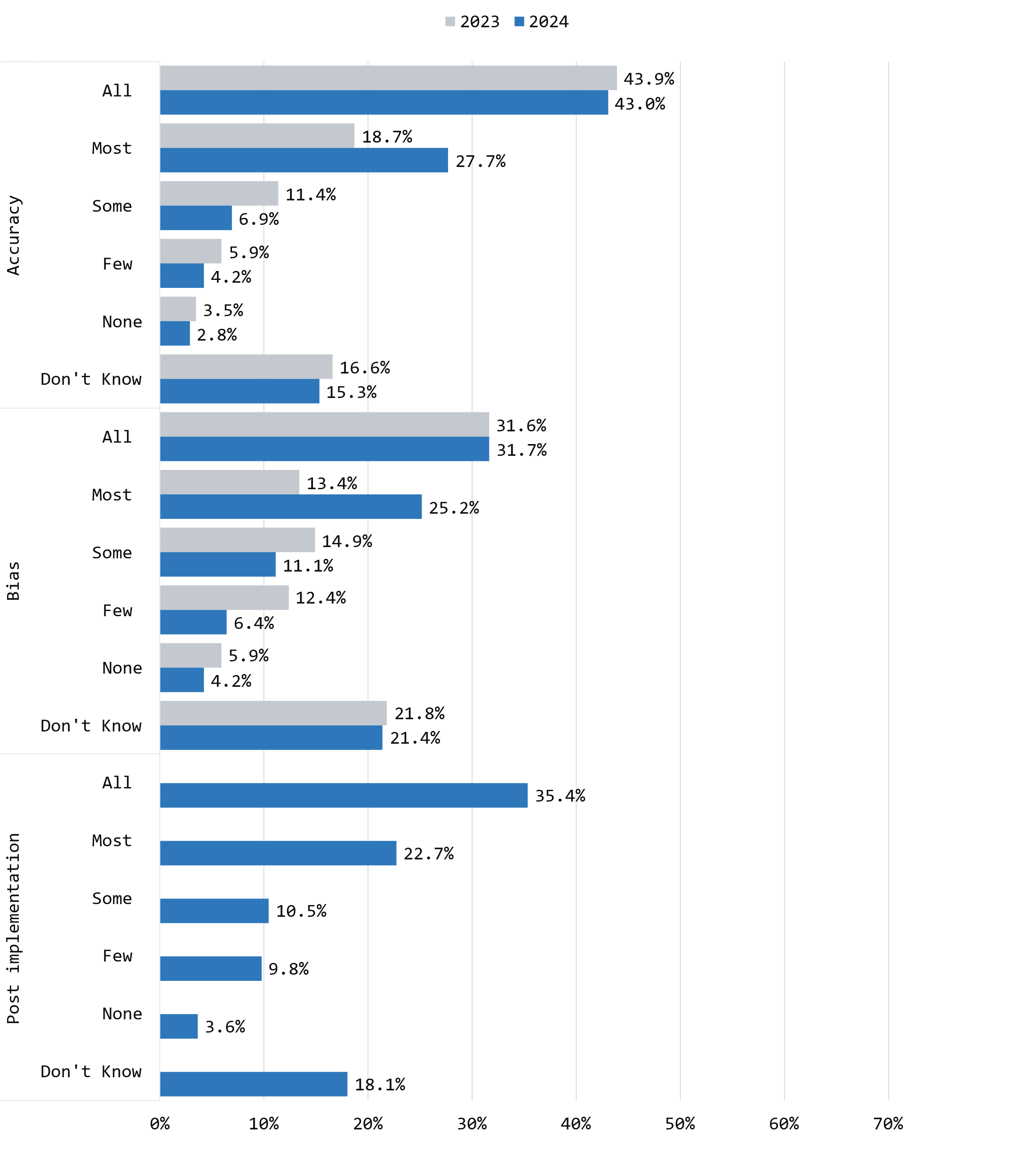 |
| Notes: Authors’ analysis of data from the 2023–2024 American Hospital Association Information Technology (IT) Supplement survey. The figure shows responses from machine-learning-adopting hospitals regarding how their models are evaluated. The question about post-implementation evaluation was only asked in the 2024 survey. Percentages are weighted by inverse probability weight (derived from propensity scores) to account for IT Supplement nonresponse. |

| **Appendix Table S1. Unweighted and Weighted Hospital Characteristics (%) by IT Supplement Response Status ^a^** | | | | |
| --- | --- | --- | --- | --- |
|  | **Nonrespondents**  **(N=3,819)** | | **Respondents**  **(N=4,730)** | |
|  | Unweighted | Weighted | Unweighted | Weighted |
| ***Organizational Context*** |  |  |  |  |
| **Hospital Type** |  |  |  |  |
| Non-Federal, Governmental | 24.5 | 21.4 | 18.5 | 21.3 |
| Not-for-Profit | 57.5 | 64.9 | 70.8 | 65.1 |
| For Profit | 18.0 | 13.7 | 10.7 | 13.6 |
| **Hospital Size** |  |  |  |  |
| Small, 0–99 beds | 58.3 | 52.3 | 47.7 | 52.3 |
| Medium, 100-399 beds | 33.9 | 36.4 | 38.9 | 36.7 |
| Large, 400 or more beds | 7.8 | 11.3 | 13.3 | 11.0 |
| **Critical Access Hospital** |  |  |  |  |
| No | 65.2 | 68.8 | 71.4 | 68.6 |
| Yes | 34.8 | 31.2 | 28.7 | 31.5 |
| **Teaching Status** |  |  |  |  |
| No | 96.4 | 94.6 | 93.3 | 94.6 |
| Yes | 3.6 | 5.4 | 6.7 | 5.4 |
| ***Environmental Context*** |  |  |  |  |
| **Health System** |  |  |  |  |
| No | 41.2 | 32.5 | 25.0 | 32.3 |
| Yes | 58.8 | 67.5 | 75.1 | 67.7 |
| **Metropolitan ^b^** |  |  |  |  |
| No | 46.0 | 41.2 | 37.9 | 41.2 |
| Yes | 54.0 | 58.8 | 62.1 | 58.8 |
| **Census Division** |  |  |  |  |
| New England | 3.9 | 3.9 | 3.7 | 3.8 |
| Middle Atlantic | 7.5 | 8.1 | 8.7 | 8.2 |
| East North Central | 16.4 | 15.9 | 14.9 | 15.9 |
| West North Central | 10.6 | 14.3 | 18.0 | 14.6 |
| South Atlantic | 12.7 | 14.8 | 16.3 | 14.7 |
| East South Central | 11.6 | 8.2 | 5.7 | 7.8 |
| West South Central | 15.3 | 14.9 | 13.6 | 14.7 |
| Mountain | 9.4 | 8.4 | 8.6 | 8.7 |
| Pacific | 12.8 | 11.6 | 10.5 | 11.6 |
| Notes: ^a^ The table compares characteristics of hospitals responding and not responding to the American Hospital Association Information Technology Supplement survey. Inverse probability weights were generated from a propensity score model that predicted the likelihood of survey response based on the hospital characteristics listed, using logistic regression. ^b^ Metropolitan status is categorized based on 2023 Rural-Urban Continuum Codes, 1-3 as metropolitan counties and 4-9 as non-metropolitan counties. | | | | |

| **Appendix Table S2. Associations Between Hospital Characteristics and Adoption of Specific Clinical Machine Learning Functions ^a^** | | | | |
| --- | --- | --- | --- | --- |
|  | **Predict Inpatient Risk** | **Predict Outpatient Risk** | **Monitor Health** | **Recommend Treatments** |
|  | **ME [95%CI]** | **ME [95%CI]** | **ME [95%CI]** | **ME [95%CI]** |
| ***Organizational Context*** |  |  |  |  |
| **Hospital Type** |  |  |  |  |
| Non-Federal, Governmental | Reference | Reference | Reference | Reference |
|  |  |  |  |  |
| Not-for-Profit | 7.5*** | 7.7*** | -0.7 | -1.9 |
|  | [3.6,11.4] | [3.4,12.0] | [-6.0,4.5] | [-6.9,3.2] |
| For Profit | -0.1 | -19.1*** | -24.7*** | -34.3*** |
|  | [-6.2,6.1] | [-25.2,-13.0] | [-30.3,-19.1] | [-40.0,-28.6] |
| **Hospital Size** |  |  |  |  |
| Small, 0–99 beds | Reference | Reference | Reference | Reference |
|  |  |  |  |  |
| Medium, 100-399 beds | 3.2 | 2.1 | -3.4 | 0 |
|  | [-0.4,6.9] | [-1.6,5.7] | [-7.5,0.6] | [-4.3,4.3] |
| Large, 400 or more beds | 12.5*** | 10.2*** | 4.2 | 3.4 |
|  | [7.2,17.7] | [5.2,15.3] | [-1.6,10.0] | [-3.0,9.9] |
| **Critical Access Hospital** | -5.1** | -4.5* | -2.3 | -3.7 |
|  | [-8.5,-1.8] | [-8.3,-0.8] | [-6.9,2.2] | [-8.5,1.1] |
| **Teaching Status** | -1.6 | -0.6 | 3.8 | 4.7 |
|  | [-9.1,5.8] | [-7.9,6.7] | [-2.2,9.8] | [-2.3,11.6] |
| ***Environmental Context*** |  |  |  |  |
| **Health System** | 22.2*** | 22.3*** | 15.9*** | 21.6*** |
|  | [19.7,24.8] | [19.2,25.4] | [10.8,21.1] | [16.9,26.4] |
| **Leading EHR ^b^** | 20.4*** | 21.7*** | 13.9*** | 14.8*** |
|  | [17.9,23.0] | [19.3,24.2] | [10.6,17.3] | [11.4,18.2] |
| **Metropolitan ^c^** | 2.7 | 2 | 4.1* | 5.3* |
|  | [-0.4,5.9] | [-1.5,5.4] | [0.1,8.2] | [1.0,9.5] |
| **Census Division** |  |  |  |  |
| New England | Reference | Reference | Reference | Reference |
|  |  |  |  |  |
| Middle Atlantic | -2.5 | 21.5*** | 7.1 | 32.0*** |
|  | [-10.5,5.5] | [10.5,32.4] | [-2.9,17.1] | [23.1,40.9] |
| East North Central | -4.7 | 17.7** | 6.4 | 19.3*** |
|  | [-12.2,2.8] | [7.0,28.4] | [-3.2,16.1] | [11.2,27.3] |
| West North Central | 4.2 | 26.9*** | -13.6** | 7.9 |
|  | [-3.1,11.4] | [16.4,37.3] | [-23.0,-4.1] | [-0.4,16.1] |
| South Atlantic | 2.4 | 25.7*** | -4.3 | 14.9*** |
|  | [-4.9,9.8] | [15.3,36.0] | [-13.8,5.3] | [6.8,22.9] |
| East South Central | -1.3 | 21.9*** | 9.9 | 14.7** |
|  | [-9.6,7.1] | [10.7,33.1] | [-1.6,21.4] | [4.6,24.7] |
| West South Central | -1.9 | 14.2** | 6.4 | 8.4 |
|  | [-9.3,5.5] | [3.5,24.9] | [-3.7,16.5] | [-0.3,17.1] |
| Mountain | 0.3 | 28.5*** | 11.3* | 23.4*** |
|  | [-7.1,7.7] | [18.1,39.0] | [1.1,21.4] | [14.7,32.1] |
| Pacific | -1.4 | 23.2*** | -2.1 | 9.8* |
|  | [-9.4,6.5] | [12.3,34.2] | [-11.8,7.7] | [1.5,18.1] |
| **Year 2024** | 1.1 | 5.7*** | -1.4 | 1.8 |
|  | [-0.7,2.9] | [3.5,7.8] | [-3.5,0.7] | [-0.3,4.0] |
| Notes: ^a^ Data are from the 2022–2023 American Hospital Association (AHA) Annual Survey and the 2023–2024 AHA IT Supplement. The sample includes 4,021 hospital-year observations. This table presents results from four separate logistic regression models, where the outcome in each model is a binary indicator for a hospital’s adoption of the specific clinical Machine Learning (ML) function. Estimates are presented as average MEs which represent the change in the probability of a specific clinical ML adoption associated with each hospital characteristic. All models are weighted using inverse propensity scores to account for IT Supplement nonresponse. MEs are scaled by 100 and can be interpreted as percentage point changes. Standard errors are clustered at the hospital level with 95% confidence intervals. ^b^ Leading EHR vendors are identified by market share. ^c^ Metropolitan status is categorized based on 2023 Rural-Urban Continuum Codes, 1-3 as metropolitan counties and 4-9 as non-metropolitan counties. Abbreviations: ME, Marginal effect. CI, confidence interval. EHR, Electronic Health Record. **P*<.05; ***P*<.01; ****P*<.001 | | | | |

| **Appendix Table S3. Associations Between Hospital Characteristics and Adoption of Specific Operational Machine Learning Functions ^a^** | | |
| --- | --- | --- |
|  | **Billing** | **Scheduling** |
|  | **ME [95%CI]** | **ME [95%CI]** |
| ***Organizational Context*** |  |  |
| **Hospital Type** |  |  |
| Non-Federal, Governmental | Reference | Reference |
|  |  |  |
| Not-for-Profit | 5.5 | 4.1 |
|  | [-0.0,11.0] | [-1.0,9.1] |
| For Profit | 0.5 | 26.4*** |
|  | [-6.2,7.2] | [19.3,33.6] |
| **Hospital Size** |  |  |
| Small, 0–99 beds | Reference | Reference |
|  |  |  |
| Medium, 100-399 beds | -0.6 | 4.6* |
|  | [-4.9,3.7] | [0.2,8.9] |
| Large, 400 or more beds | 6.8* | 11.6*** |
|  | [0.3,13.3] | [5.3,18.0] |
| **Critical Access Hospital** | -4.5 | -4.7* |
|  | [-9.7,0.7] | [-9.3,-0.1] |
| **Teaching Status** | -3.4 | 0.5 |
|  | [-10.9,4.1] | [-7.0,8.0] |
| ***Environmental Context*** |  |  |
| **Health System** | 17.8*** | 20.3*** |
|  | [12.7,22.8] | [15.6,25.1] |
| **Leading EHR ^b^** | 16.6*** | 29.7*** |
|  | [13.2,20.0] | [26.7,32.7] |
| **Metropolitan ^c^** | 2.9 | 0.9 |
|  | [-1.6,7.3] | [-3.1,4.8] |
| **Census Division** |  |  |
| New England | Reference | Reference |
|  |  |  |
| Middle Atlantic | 13.2** | 15.8*** |
|  | [4.1,22.2] | [6.5,25.1] |
| East North Central | 17.1*** | 12.4** |
|  | [8.6,25.7] | [3.8,21.0] |
| West North Central | 21.1*** | 19.5*** |
|  | [12.5,29.7] | [10.9,28.1] |
| South Atlantic | 18.3*** | 18.9*** |
|  | [9.9,26.6] | [10.4,27.5] |
| East South Central | 10.6* | 7.3 |
|  | [0.6,20.6] | [-2.8,17.5] |
| West South Central | 12.2** | 13.9** |
|  | [3.2,21.1] | [4.9,23.0] |
| Mountain | 23.0*** | 13.2** |
|  | [14.2,31.8] | [3.7,22.7] |
| Pacific | 34.2*** | 13.9** |
|  | [25.1,43.2] | [4.8,23.1] |
| **Year 2024** | 20.0*** | 14.1*** |
|  | [17.6,22.4] | [11.9,16.3] |
| Notes: ^a^ Data are from the 2022–2023 American Hospital Association (AHA) Annual Survey and the 2023–2024 AHA Information Technology (IT) Supplement. The sample includes 4,021 hospital-year observations. This table presents results from two separate logistic regression models, where the outcome in each model is a binary indicator for a hospital’s adoption of the specific operational machine learning (ML). Estimates are presented as average MEs which represent the change in the probability of an operational ML adoption associated with each hospital characteristic. All models are weighted using inverse propensity scores to account for IT Supplement nonresponse. MEs are scaled by 100 and can be interpreted as percentage-point changes. Standard errors are clustered at the hospital level. ^b^ Leading EHR vendors are identified by market share. ^c^ Metropolitan status is categorized based on 2013 Rural-Urban Continuum Codes, 1-3 as metropolitan counties and 4-9 as non-metropolitan counties. Abbreviations: ME, Marginal effect. CI, confidence interval. EHR, Electronic Health Record. **P*<.05; ***P*<.01; ****P*<.001 | | |

| **Appendix Table S4. Sensitivity Analysis of Associations Between Hospital Characteristics and Machine Learning Adoption without Applying Inverse Probability Weight ^a^** | | | |
| --- | --- | --- | --- |
|  | **Marginal Effects** | **95% CI** | ***P*-Value** |
| ***Organizational Context*** |  |  |  |
| **Hospital Type** |  |  |  |
| Non-Federal, Governmental | Reference |  |  |
| Not-for-Profit | 5.4** | [2.0,8.9] | .002 |
| For Profit | -6.5* | [-12.4,-0.5] | .03 |
| **Hospital Size** |  |  |  |
| Small, 0–99 beds | Reference |  |  |
| Medium, 100-399 beds | 2.8 | [-0.5,6.2] | .10 |
| Large, 400 or more beds | 13.4*** | [8.5,18.2] | <.001 |
| **Critical Access Hospital** | -6.5*** | [-9.9,-3.0] | <.001 |
| **Teaching Status** | -2.3 | [-11.5,6.9] | .62 |
| ***Environmental Context*** |  |  |  |
| **Health System** | 25.2*** | [21.0,29.4] | <.001 |
| **Leading EHR ^b^** | 18.5*** | [15.4,21.6] | <.001 |
| **Metropolitan ^c^** | 4.4** | [1.4,7.5] | .004 |
| **Census Division** |  |  |  |
| New England | Reference |  |  |
| Middle Atlantic | -2.1 | [-9.6,5.4] | .58 |
| East North Central | 0.1 | [-6.9,7.0] | .99 |
| West North Central | 5.9 | [-0.8,12.6] | .09 |
| South Atlantic | 8.3* | [1.5,15.1] | .02 |
| East South Central | 1.8 | [-6.1,9.6] | .66 |
| West South Central | -1.3 | [-8.3,5.7] | .72 |
| Mountain | 6.0 | [-0.8,12.8] | .09 |
| Pacific | -2.7 | [-10.2,4.9] | .49 |
| **Year 2024** | 1.8* | [0.2,3.4] | .03 |
| Notes: ^a^ This table presents a sensitivity analysis of the main model on any machine learning (ML) adoption without applying the inverse probability weights. Data are from the 2022–2023 American Hospital Association (AHA) Annual Survey and the 2023–2024 AHA Information Technology (IT) Supplement. The sample includes 4,055 hospital-year observations. The table reports average marginal effects (MEs) from a logistic regression. MEs are scaled by 100 and can be interpreted as percentage point changes. Standard errors are clustered at the hospital level. ^b^ Leading EHR vendors are identified by market share. ^c^ Metropolitan status is categorized based on 2023 Rural-Urban Continuum Codes, 1-3 as metropolitan counties and 4-9 as non-metropolitan counties. Abbreviations: EHR, Electronic Health Record. CI, confidence interval. **P*<.05; ***P*<.01; ****P*<.001 | | | |

| **Appendix Table S5. Sensitivity Analysis of Associations Between Hospital Characteristics and Types of ML Adoption in HER without Applying Inverse Probability Weight ^a^** | | | |
| --- | --- | --- | --- |
|  | **Marginal Effects** | **95% CI** | ***P*-Value** |
| **PANEL A: CLINICAL ML ONLY** |  |  |  |
| ***Organizational Context*** |  |  |  |
| **Hospital Type** |  |  |  |
| Non-Federal, Governmental | Reference |  |  |
| Not-for-Profit | -0.3 | [-4.8,4.2] | .89 |
| For Profit | -15.4*** | [-20.2,-10.7] | <.001 |
| **Hospital Size** |  |  |  |
| Small, 0–99 beds | Reference |  |  |
| Medium, 100-399 beds | 0 | [-3.6,3.7] | .98 |
| Large, 400 or more beds | 2.5 | [-3.4,8.4] | .40 |
| **Critical Access Hospital** | 3.1 | [-1.1,7.2] | .15 |
| **Teaching Status** | -6.5** | [-11.3,-1.6] | .009 |
| ***Environmental Context*** |  |  |  |
| **Health System** | 3.7 | [-0.2,7.6] | .06 |
| **Leading EHR ^b^** | -3.7* | [-6.9,-0.5] | .03 |
| **Metropolitan ^c^** | -0.3 | [-3.7,3.2] | .88 |
| **Census Division** |  |  |  |
| New England | Reference |  |  |
| Middle Atlantic | -9.8* | [-18.8,-0.8] | .03 |
| East North Central | -18.6*** | [-26.8,-10.5] | <.001 |
| West North Central | -9.8* | [-18.4,-1.3] | .02 |
| South Atlantic | -17.1*** | [-25.4,-8.8] | <.001 |
| East South Central | 0.3 | [-9.8,10.4] | .95 |
| West South Central | -14.5*** | [-23.0,-5.9] | <.001 |
| Mountain | -13.8** | [-22.6,-5.0] | .002 |
| Pacific | -21.0*** | [-29.3,-12.7] | <.001 |
| **Year 2024** | -10.0*** | [-12.0,-8.0] | <.001 |
| **PANEL B: OPERATIONAL ML ONLY** |  |  |  |
| ***Organizational Context*** |  |  |  |
| **Hospital Type** |  |  |  |
| Non-Federal, Governmental | Reference |  |  |
| Not-for-Profit | 0.4 | [-0.7,1.5] | .47 |
| For Profit | -0.4 | [-1.8,0.9] | .52 |
| **Hospital Size** |  |  |  |
| Small, 0–99 beds | Reference |  |  |
| Medium, 100-399 beds | 0.2 | [-0.8,1.3] | .64 |
| Large, 400 or more beds | -1.1* | [-2.1,-0.2] | .02 |
| **Critical Access Hospital** | -0.5 | [-1.6,0.6] | .40 |
| **Teaching Status** | 1.5 | [-1.7,4.8] | .36 |
| ***Environmental Context*** |  |  |  |
| **Health System** | -2.6** | [-4.6,-0.6] | .01 |
| **Leading EHR** | 0.6 | [-0.1,1.3] | .08 |
| **Metropolitan** | 0 | [-0.9,0.9] | .97 |
| **Census Division** |  |  |  |
| New England | Reference |  |  |
| Middle Atlantic | -0.7 | [-2.2,0.7] | .33 |
| East North Central | 1.5 | [-0.2,3.3] | .09 |
| West North Central | 0.2 | [-1.6,2.0] | .83 |
| South Atlantic | 1.8* | [0.0,3.7] | .05 |
| East South Central | 0 | [-1.9,1.9] | .97 |
| West South Central | -0.1 | [-1.8,1.6] | .88 |
| Mountain | 0 | [-1.8,1.8] | .995 |
| Pacific | -1 | [-2.3,0.4] | .17 |
| **Year 2024** | 1.8*** | [1.1,2.4] | <.001 |
| **PANEL C: Both Types** |  |  |  |
| ***Organizational Context*** |  |  |  |
| **Hospital Type** |  |  |  |
| Non-Federal, Governmental | Reference |  |  |
| Not-for-Profit | 6.1* | [1.0,11.1] | .02 |
| For Profit | 10.0** | [3.0,17.0] | .005 |
| **Hospital Size** |  |  |  |
| Small, 0–99 beds | Reference |  |  |
| Medium, 100-399 beds | 2.4 | [-1.8,6.7] | .26 |
| Large, 400 or more beds | 12.0*** | [5.6,18.4] | <.001 |
|  |  |  |  |
| **Critical Access Hospital** | -9.1*** | [-13.9,-4.3] | <.001 |
| **Teaching Status** | 3.1 | [-5.3,11.6] | .47 |
| ***Environmental Context*** |  |  |  |
| **Health System** | 24.4*** | [19.7,29.1] | <.001 |
| **Leading EHR** | 21.7*** | [18.0,25.5] | <.001 |
| **Metropolitan** | 4.8* | [0.8,8.9] | .02 |
| **Census Division** |  |  |  |
| New England | Reference |  |  |
| Middle Atlantic | 8.8 | [-1.0,18.6] | .08 |
| East North Central | 17.6*** | [8.3,26.9] | <.001 |
| West North Central | 15.7*** | [6.4,25.1] | .001 |
| South Atlantic | 23.8*** | [14.5,33.1] | <.001 |
| East South Central | 1.8 | [-9.0,12.6] | .74 |
| West South Central | 13.7** | [4.1,23.4] | .005 |
| Mountain | 20.1*** | [10.2,30.0] | <.001 |
| Pacific | 19.9*** | [10.1,29.7] | <.001 |
| **Year 2024** | 9.8*** | [7.6,12.0] | <.001 |
| Notes: ^a^ Data are from the 2022–2023 American Hospital Association (AHA) Annual Survey linked with the 2023–2024 AHA Information Technology (IT) Supplement. The sample includes 4,055 hospital-year observations. The table presents average marginal effects (MEs) from a single multinomial logistic regression, where the outcome is a four-category measure of machine learning (ML) adoption type: clinical only (Panel A), operational only (Panel B), both (Panel C), and no ML adoption (the base outcome). Each ME represents the change in probability of being in a specific category associated with a hospital characteristic. The model is weighted using inverse probability weight (derived from propensity scores) to account for IT Supplement nonresponse. MEs are scaled by 100 and can be interpreted as percentage point changes. Standard errors are clustered at the hospital level to account for within hospital correlation over time. ^b^ Leading EHR vendors are identified by market share. ^c^ Metropolitan status is categorized based on 2023 Rural-Urban Continuum Codes, 1-3 as metropolitan counties and 4-9 as non-metropolitan counties. Abbreviations: ML, Machine Learning, EHR, Electronic Health Record. CI, confidence interval. **P*<.05; ***P*<.01; ****P*<.001 | | | |

| **Appendix Table S6. Associations Between Hospital Characteristics and Machine Learning Adoption, Separately for Small and Medium Hospitals ^a^** | | | | | | |
| --- | --- | --- | --- | --- | --- | --- |
|  | **Small, 0–99 beds** | | | **Medium, 100-399 beds** | | |
|  | **Marginal Effects** | **95% CI** | ***P*-Value** | **Marginal Effects** | **95% CI** | ***P*-Value** |
| ***Organizational Context*** |  |  |  |  |  |  |
| **Hospital Type** |  |  |  |  |  |  |
| Non-Federal, Governmental | Reference |  |  | Reference |  |  |
| Not-for-Profit | 4.0 | [-1.7,9.8] | .17 | 6.5 | [-0.0,13.0] | .05 |
| For Profit | -12.2* | [-23.2,-1.1] | .03 | -5.8 | [-15.5,4.0] | .25 |
| **Critical Access Hospital** | -9.2*** | [-13.8,-4.7] | <.001 | -11.7** | [-20.1,-3.3] | .006 |
| **Teaching Status** | N/A ^b^ | N/A |  | -3.0 | [-15.5,9.4] | .63 |
| ***Environmental Context*** |  |  |  |  |  |  |
| **Health System** | 25.6*** | [21.7,29.5] | <.001 | 17.3*** | [12.9,21.6] | <.001 |
| **Leading EHR ^c^** | 23.6*** | [19.1,28.1] | <.001 | 14.8*** | [10.3,19.4] | <.001 |
| **Metropolitan ^d^** | 5.0 | [-0.2,10.1] | .06 | 4.6 | [-0.2,9.5] | .06 |
| **Census Division** |  |  |  |  |  |  |
| New England | Reference |  |  | Reference |  |  |
| Middle Atlantic | -1.6 | [-18.4,15.1] | .85 | -5.1 | [-15.4,5.3] | .34 |
| East North Central | 2.0 | [-10.5,14.4] | .76 | 2 | [-8.8,12.8] | .72 |
| West North Central | 9.7 | [-2.5,21.9] | .12 | 4.9 | [-6.2,15.9] | .39 |
| South Atlantic | 10.9 | [-2.4,24.2] | .11 | 8.9 | [-0.8,18.5] | .07 |
| East South Central | 6.9 | [-8.2,22.1] | .37 | 1.8 | [-10.0,13.6] | .77 |
| West South Central | -4.1 | [-17.0,8.8] | .53 | 2.6 | [-8.2,13.4] | .64 |
| Mountain | 7.5 | [-5.0,20.0] | .24 | 12.5* | [1.7,23.3] | .02 |
| Pacific | 6.6 | [-7.4,20.5] | .36 | -6.9 | [-17.7,3.9] | .21 |
| **Year 2024** | 2.3 | [-0.8,5.3] | .14 | 1.4 | [-1.3,4.1] | .31 |
| **Number of Observations** | 1,800 |  |  | 1,662 |  |  |
| Notes: ^a^ This table presents a sensitivity analysis of the main model on any machine learning adoption without applying the inverse probability weights. Data are from the 2022–2023 American Hospital Association (AHA) Annual Survey and the 2023–2024 AHA IT Supplement. The table reports average marginal effects (MEs) from a logistic regression where the outcome is any machine learning adoption. Marginal effects are scaled by 100 and can be interpreted as percentage point changes. Standard errors are clustered at the hospital level. ^b^ Estimates are infeasible due to lack of variation. ^c^ Leading EHR vendors are identified by market share. ^d^ Metropolitan status is categorized based on 2023 Rural-Urban Continuum Codes, 1-3 as metropolitan counties and 4-9 as non-metropolitan counties. Abbreviations: EHR, Electronic Health Record. CI, confidence interval. **P*<.05; ***P*<.01; ****P*<.001 | | | | | | |
